# Relaxometry and brain myelin quantification with synthetic MRI in MS subtypes and their associations with spinal cord atrophy

**DOI:** 10.1016/j.nicl.2022.103166

**Published:** 2022-08-25

**Authors:** Theodoros Ladopoulos, Britta Matusche, Barbara Bellenberg, Florian Heuser, Ralf Gold, Carsten Lukas, Ruth Schneider

**Affiliations:** a Department of Neurology, St. Josef Hospital, Ruhr-University Bochum, Gudrunstr. 56, 44791 Bochum, Germany; b Institute of Neuroradiology, St. Josef Hospital, Ruhr-University Bochum, Gudrunstr. 56, 44791 Bochum, Germany; c Department of Diagnostic and Interventional Radiology and Nuclear Medicine, St. Josef Hospital, Ruhr-University Bochum, Gudrunstr. 56, 44791 Bochum, Germany

**Keywords:** BPF, brain parenchymal fraction,, CSF, cerebrospinal fluid, EDSS, expanded disability status scale, GM, grey matter, GMF, grey matter fraction, CS, control subjects, DGM, deep grey matter, ICV, intracranial volume, MS, Multiple sclerosis, MUCCA, mean upper cervical cord area, MVF, myelin volume fraction, iMVF, intracranial myelin volume fraction, aMVF, average myelin volume fraction, MRI, magnetic resonance imaging, NAWM, normal appearing white matter, PMS, progressive multiple sclerosis, PPMS, primary progressive multiple sclerosis, QRAPMASTER, quantification of relaxation times and proton density by multi-echo acquisition of a saturation recovery using turbo spin-echo readout, ROI, region of interest, RRMS, relapsing remitting multiple sclerosis, SPMS, secondary progressive multiple sclerosis, WM, white matter, WMF, white matter fraction, Multiple Sclerosis, MRI, Myelin imaging, Relaxation times, Spinal cord atrophy, Synthetic MR

## Abstract

Immune-mediated demyelination and neurodegeneration are pathophysiological hallmarks of Multiple Sclerosis (MS) and main drivers of disease related disability. The principal method for evaluating qualitatively demyelinating events in the clinical context is contrast-weighted magnetic resonance imaging (MRI). Moreover, advanced MRI sequences provide reliable quantification of brain myelin offering new opportunities to study tissue pathology in vivo. Towards neurodegenerative aspects of the disease, spinal cord atrophy – besides brain atrophy – is a powerful and validated predictor of disease progression. The etiology of spinal cord volume loss is still a matter of research, as it remains unclear whether the impact of local lesion pathology or the interaction with supra- and infratentorial axonal degeneration and demyelination of the long descending and ascending fiber tracts are the determining factors.

Quantitative synthetic MR using a multiecho acquisition of saturation recovery pulse sequence provides fast automatic brain tissue and myelin volumetry based on R1 and R2 relaxation rates and proton density quantification, making it a promising modality for application in the clinical routine. In this cross sectional study a total of 91 MS patients and 31 control subjects were included to investigate group differences of global and regional measures of brain myelin and relaxation rates, in different MS subtypes, using QRAPMASTER sequence and SyMRI postprocessing software. Furthermore, we examined associations between these quantitative brain parameters and spinal cord atrophy to draw conclusions about possible pathophysiological relationships.

Intracranial myelin volume fraction of the global brain exhibited statistically significant differences between control subjects (10.4%) and MS patients (RRMS 9.4%, PMS 8.1%). In a LASSO regression analysis with total brain lesion load, intracranial myelin volume fraction and brain parenchymal fraction, the intracranial myelin volume fraction was the variable with the highest impact on spinal cord atrophy (standardized coefficient 4.52). Regional supratentorial MRI metrics showed altered average myelin volume fraction, R1, R2 and proton density in MS patients compared to controls most pronounced in PMS. Interestingly, quantitative MRI parameters in supratentorial regions showed strong associations with upper cord atrophy, suggesting an important role of brain diffuse demyelination on spinal cord pathology possibly in the context of global disease activity. R1, R2 or proton density of the thalamus, cerebellum and brainstem correlated with upper cervical cord atrophy, probably reflecting the direct functional connection between these brain structures and the spinal cord as well as the effects of retrograde and anterograde axonal degeneration.

By using Synthetic MR-derived myelin volume fraction, we were able to effectively detect significant differences of myelination in relapsing and progressive MS subtypes. Total intracranial brain myelin volume fraction seemed to predict spinal cord volume loss better than brain atrophy or total lesion load. Furthermore, demyelination in highly myelinated supratentorial regions, as an indicator of diffuse disease activity, as well as alterations of relaxation parameters in adjacent infratentorial and midbrain areas were strongly associated with upper cervical cord atrophy.

## Highlights

• Synthetic MR was able to quantify differences on MVF, R1, R2 and PD between patients with MS and CS.
• Intracranial MVF has a greater impact on spinal cord volume loss compared to BPF or total brain lesion load.
• MUCCA was associated with supratentorial demyelination as a result of diffuse disease activity.
• Alterations in R1, R2 and PD in adjacent infratentorial ROIs were associated with spinal cord atrophy.

## 1 Introduction

Demyelination is one of the leading pathophysiological mechanisms in Multiple Sclerosis (MS) contributing to physical disability as well as cognitive deficits (Kolind et al., 2012, Campanholo et al., 2022). It is well known that conventional contrast-weighted MRI sequences lack histopathological specificity and sensitivity, thus failing to detect diffuse inflammatory demyelinating processes in normal appearing white (NAWM) and grey matter (Moll et al., 2011). Furthermore, a differentiation between the degree of concurrent edema, axonal injury and demyelination is not possible regarding conventional T2 lesions (Tillema and Pirko, 2013).

There have been many endeavors in the last 20 years to succeed accurate quantification of myelin content in the brain tissue by using advanced quantitative MRI methods. Some of the most established MRI methods for myelin imaging are myelin water imaging, T1/T2 ratio and magnetization transfer imaging (Heath et al., 2018). Drawbacks of these techniques include relatively long scanning time as well as limited sensitivity to demyelination, questioning their applicability in the clinical setting. A recently developed quantitative imaging technique termed QRAPMASTER (quantification of relaxation times and proton density by multi-echo acquisition of a saturation recovery using turbo spin-echo readout) allows the estimation of longitudinal (R1) and transversal (R2) relaxation rates and proton density (PD) from a single pulse sequence. Relaxation times, as it is widely known in the literature, correlate nonspecifically with tissue pathology, i.e. demyelination, edema or axonal injury, in MS patients (MacKay et al., 2009, Deoni, 2010). Furthermore QRAPMASTER allows estimation of myelin in the brain tissue (Warntjes et al., 2007, Warntjes et al., 2008, Warntjes et al., 2016), which correlated well with tissue myelin stained with luxol fast blue and proteolipid protein immunostaining in two previous histopathological studies, supporting the validity of the technique (Warntjes et al., 2017, Ouellette et al., 2020). Furthermore, a recent study demonstrated significant associations of whole brain and normal appearing white matter myelin fractions with clinical (expanded disability status scale, EDSS) and cognitive disability measures in relapsing remitting (RRMS) and progressive MS (PMS) patients (Ouellette et al., 2020). Nevertheless, it is well known that not only inflammation and demyelination but also neurodegenerative processes are also of great importance regarding accumulation of disability, however whether or not neurodegeneration is driven by inflammation or demyelination is still controversial discussed (Peterson and Fujinami, 2007).

Besides brain atrophy, upper cervical cord atrophy has been reliably used to monitor disease progression in MS as a measure of neurodegeneration and axonal loss (Lukas et al., 2015). Spinal cord atrophy differs among MS-subgroups regarding the time of onset, the rate of progression and the extent of cervical cord atrophy. Patients with primary progressive (PPMS) and secondary progressive (SPMS) demonstrate more severe cervical cord volume changes than RRMS patients (Bernitsas et al., 2015, Mina et al., 2021). Although spinal cord atrophy correlates robustly with common disability scales (EDSS, MSFC), its specific etiology remains to be deciphered, since it is only partially dependent on the presence of local MS lesions within the cervical cord (Evangelou et al., 2005, Rocca et al., 2011). It has been hypothesized that anterograde and retrograde axonal degeneration in long fiber tracts of the brain subsequent to demyelination and neuroaxonal injury may play a determining role in the pathogenesis of cervical cord atrophy (Lukas et al., 2013, Bellenberg et al., 2015). Considering this hypothesis, quantitative MRI techniques, like QRAPMASTER, could shed some light on the origins of spinal cord atrophy by investigating linkages with quantitative measures of white matter pathology of the brain.

The aim of this study was to compare global and regional myelin measures and relaxation rates derived from the QRAPMASTER sequence between MS groups and control subjects and to investigate associations of those parameters in several infra- und supratentorial brain structures with the mean upper cervical cord area (MUCCA) in MS patients. We hypothesized that brain myelin loss may show a closer relation to MUCCA loss than brain atrophy and total brain lesion load. Furthermore, we assumed that associations between cervical cord volume and brain structures will be most pronounced in anatomically connected structures, such as the thalamus, brainstem, cerebellum and the corticospinal and spinocerebellar tracts, and that associations between upper cervical cord volume and brain myelin might differ between progressive and relapsing forms of MS.

## 2 Methods

### 2.1 Study participants, demographical data and clinical parameters

MRI and clinical data from 49 RRMS, 27 SPMS, 15 PPMS and 31 control subjects (CS) were acquired from November 2018 till December 2019 in the context of routine clinical follow up and were retrospectively reviewed in a cross-sectional manner. Patients had to fulfill definitive diagnosis of MS according to 2017 McDonald criteria (Thompson et al., 2018) with an age of under 65 years. In addition, we included control subjects with no neurological deficit who have been scanned as part of clinical routine MRI examinations in order to rule out any intracranial pathology. Exclusion criteria were: other intracranial pathology, e.g. small vessel disease, ischemic or hemorrhagic stroke, hydrocephalus or tumor, as well as bad or marginal MRI image quality. 9 MRI scans were excluded because of motion or swallowing artifacts. Demographical data (age, sex, disease duration) were collected from the electronic health record system of our hospital. The EDSS was acquired by experienced neurologists. The study protocol was approved by the ethics committee of the Medical Faculty of the Ruhr-University Bochum (Approval Np. 20-7054-BR).

### 2.2 MRI acquisition

All MR imaging sequences were performed on a 1.5 T scanner (Aera, Siemens Healthineers, Erlangen, Germany) using a 16 channel head/neck matrix coil. All patients underwent quantitative MR imaging using the QRAPMASTER sequence (repetition time: 6930 ms, echo time 1: 23 ms, echo time 2: 102 ms, inversion time: 29 ms, acquisition matrix: 256x146, voxel size: 1x1x4 mm^3^) and conventional contrast weighted imaging including sagittal 3D T1-weighted (T1w) MPRAGE (repetition time: 10 ms, echo time: 4.6 ms, inversion time: 1000 ms, flip angle 8°, acquisition matrix: 240x240, voxel size: 1x1x1 mm^3^, 180 slices) and sagittal 3D FLAIR (repetition time/echo time/inversion time: 5000 ms / 332 ms / 1800 ms, flip angle 120°, number of excitations:1, voxel size 1 × 1 × 1 mm^3^, matrix: 256 × 230, 160 slices) images of the brain. MRI sequences with contrast enhancement using gadolinium were always performed after the acquisition of QRAPMASTER sequence to avoid changes of relaxation rates and possible interference with myelin quantification by virtue of blood brain barrier breakdown and paramagnetic effects of contrast agents (Blystad et al., 2016).

The herein used QRAPMASTER sequence for quantitative imaging is a multislice, multiecho, multisaturation delay acquisition sequence that allows simultaneous time efficient quantification of T1, T2 relaxation times and PD. The original technique was described thoroughly in previous work(Warntjes et al., 2007, Warntjes et al., 2008). Briefly, the sequence consists of an interleaved saturation pulse (block 1) and a Carr-Purcell-Meiboom-Gill acquisition (block 2) acting on two different slices for the independent estimation of T1 and T2 relaxation times, and the local B1 field. Longitudinal and transverse relaxation rates (R1 and R2) are calculated by inverting T1 and T2 times. The use of echo planar imaging technique and a saturation instead of an inversion recovery pulse in a multi-slice acquisition context succeeds tissue relaxometry and estimation of PD in less than seven minutes.

### 2.3 Image analysis

#### 2.3.1 Quantification of global brain measures

**Fig. 1:**
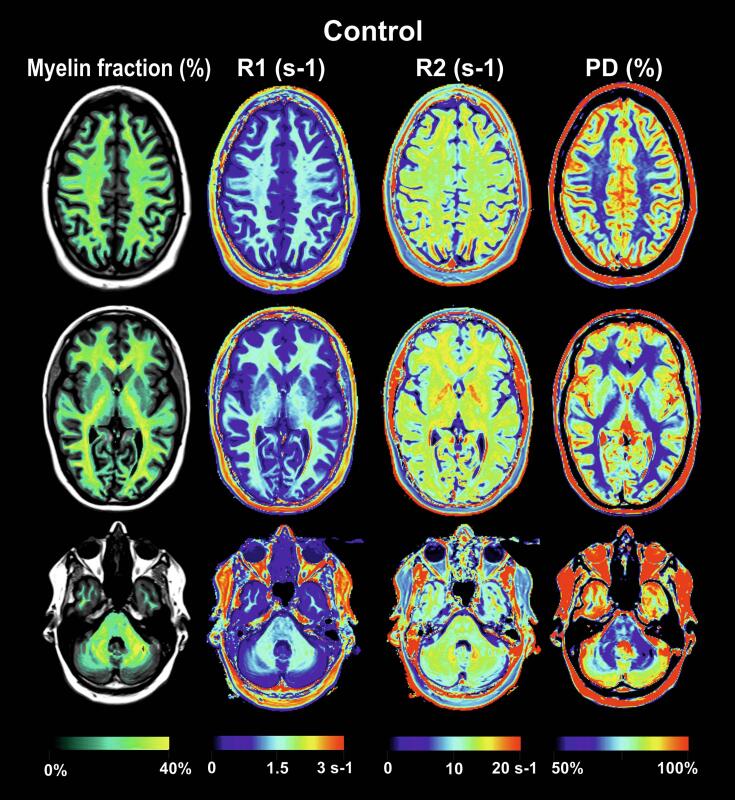
SyMRI-derived maps of myelin volume fraction, relaxations rates R1 and R2, proton density of a control subject.

**Supplementary figure 1:**
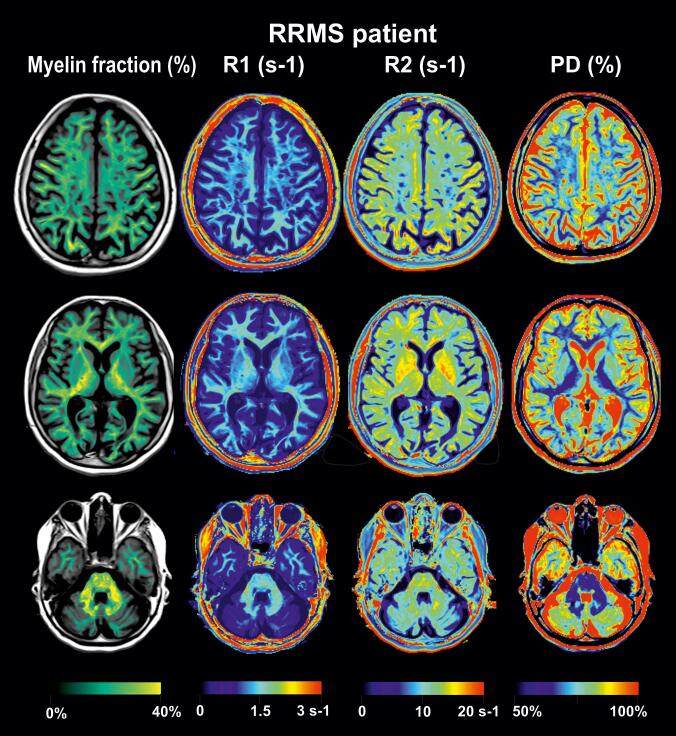


The brain segmentation into white matter (WM), gray matter (GM) and cerebrospinal fluid (CSF) is based on a tissue look up grid in a three dimensional R1-R2-PD space which has been created on the basis of T1 and T2 relaxometry values in different brain regions of healthy volunteers(West et al., 2012). Myelin volume fraction is calculated using a 4-partial-volume-compartment hypothesis composed of the cellular and extracellular brain microstructure; the myelin partial volume, cellular partial volume, free water partial volume, and excess parenchymal water partial volume. According to this model the relaxation of each compartment contributes to the effective relaxation of an acquired voxel. Taking into account the magnetization exchange rates, the relaxation rates and the proton density of these four compartments it is possible to calculate the myelin tissue fraction in each voxel (Warntjes et al., 2016). Using the SyMRI® Software (Version 11.1.5 for Windows, Synthetic MR, Linköping, Sweden) total brain myelin, GM and WM as well as CSF were extracted as absolute volumes in milliliter and as fractions of the intracranial volume (ICV). Myelin as a fraction of ICV (iMVF, intracranial MVF = total brain myelin volume/intracranial volume) was used as a normalized measure of brain myelin in our analysis to account for physiological sex and body-size related differences. Additionally, mean values of R1 (s^−1^), R2 (s^−1^) and PD (%) within the global brain were calculated. Fig. 1 depicts maps of relaxation rates, PD and myelin of a control subject. A typical example of an RRMS patient is included in the supplementary material (Fig. S1).

To extract the global brain lesion load we generated binary MS lesion maps based on conventional 3D FLAIR images using the lesion prediction algorithm (Schmidt et al., 2019) as implemented in the LST toolbox (version 2.0.15; https://www.statistical-modelling.de/lst.html) for Statistical Parametric Mapping (SPM.12; https://www.fil.ion.ucl.ac.uk/spm/software/spm12, University College London, London, UK). Furthermore, we have thoroughly inspected lesion maps for misclassification and manually edited them using the FSLeyes viewer if necessary.

#### 2.3.2 Region-of-interest-based quantification of average MVF, R1, R2 and PD

**Fig. 2:**
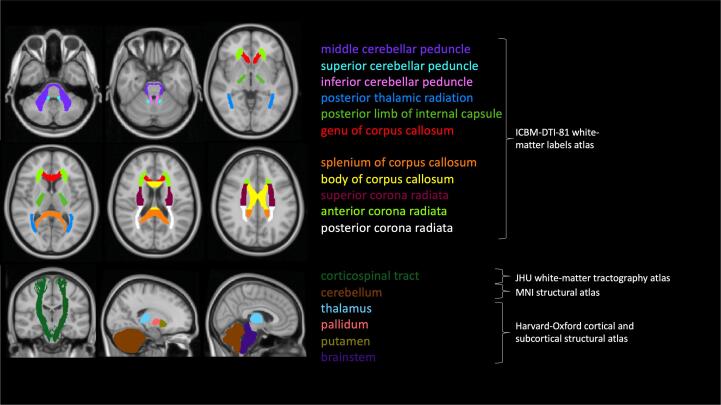
Selected regions of interest and the corresponding anatomical atlases.

Four different atlases for brain structures (ICBM-DTI-81 atlas, Harvard-Oxford cortical and subcortical structural atlas, MNI structural atlas, JHU white matter tractography atlas) were used to extract average MVF, R1, R2 and PD fractions in 28 brain regions of interests (ROI). The average MVF (aMVF) represents the mean value of MVF in all ROI-voxels. Since our main interest was to study associations between cervical cord area and quantitative brain measures we focused on brain ROIs which are functionally or structurally connected to the spinal cord (Harrow-Mortelliti et al., (FL)2021.). We analyzed the corticospinal tracts (CST, including the entire course of the fiber tracts from corona radiate to the cerebral peduncles), internal capsule (especially the posterior part), corona radiata, deep gray matter (putamen, globus pallidus), thalamus, cerebellar peduncles, cerebellum and brainstem. Additionally, we included splenium, body and genu of corpus callosum as highly myelinated reference structures with typical inflammatory demyelinating lesions in a great percentage of MS patients (Evangelou et al., 2000, Larson et al., 2002). Fig. 2 illustrates the selected regions and the underlying anatomical atlases.

**Fig. 3:**
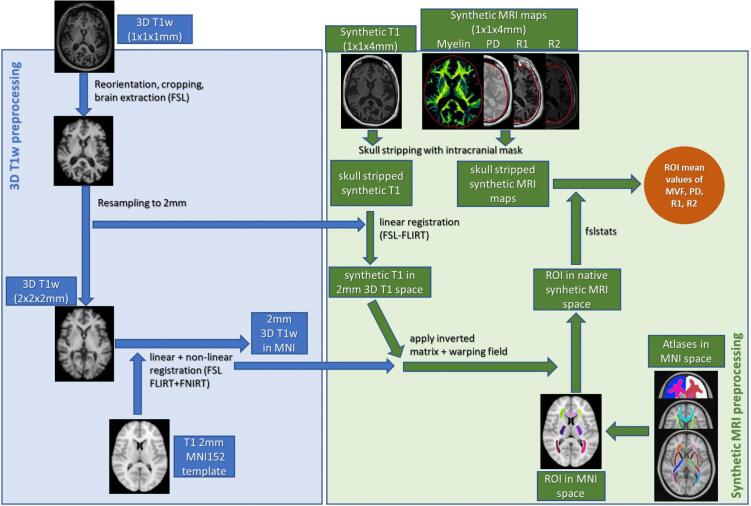
Schematic description of the processing of the MRI data in FSL.

We established an analysis pipeline using FSL software tools (https://fsl.fmrib.ox.ac.uk/fsl/fslwiki) for image registration among the different MRI sequences and for the extraction of average quantitative MRI values within ROIs (Fig. 3) (Jenkinson et al., 2012). In summary, the SyMRI software automatically performs skull stripping on myelin maps and generates an intracranial mask which represents the sum of brain tissue and CSF, removing other tissue elements. Using fslmaths, the intracranial mask was applied to the synthetic T1w images and to R1, R2 and PD maps to carry out skull stripping. For the 3D T1w MPRAGE images, reorientation, cropping, and brain extraction was first performed using FSL. They were then resampled to an isotropic voxel size of 2 mm to bridge the difference to the synthetic MRI images (voxel size of 1 x1 × 4 mm^3^) which were linearly registered to the resampled 3D T1w images (flirt). The 3D T1w images were then registered to the FSL’s 2 mm MNI template using linear and non-linear registration (flirt + fnirt). The obtained warping fields were inverted in order to register each ROI mask in MNI space to individual 3D T1w space. Likewise, the matrices from the registration of synthetic T1w to 3D T1w images were inverted and applied to ROI masks in 3D T1w space in order to obtain ROI masks in individual synthetic MRI space. Mean values and standard deviations of aMVF, R1, R2, and PD within each ROI were extracted using fslstats.

Using the same functions we estimated the global lesion load volume extracted from 3D FLAIR series after proper registration on the 3D-T1w images.

#### 2.3.3 Quantification of the cervical cord area

For the assessment of spinal cord volumes, measurements of the mean upper cervical cord area (MUCCA) were performed based on the sagittal 3D-T1w image series of the brain. MUCCA was measured using the semi-automated software NeuroQLab (Version 4.01, Fraunhofer MEVIS, Bremen, Germany) over a predefined cord section with a length of 30 mm (starting from the top of the C1 vertebra) as described in previous works(Lukas et al., 2013). In brief, the method fits a Gaussian mixture model to the intensity histogram of the segmented upper cervical cord region controlling for partial volume effects. Prior to the quantification step, cervical cord lesions appearing hypointense on T1 weighting (“black holes”) were filled by a simple morphologic technique as they could lead to distorted cord segmentations. MUCCA was calculated by dividing the segmented cord volume by the section length yielding the average cord area at the C1–C3 vertebral level.

### 2.4 Statistical analysis

SPSS software (IBM Corp. Released 2016. IBM SPSS Statistics for Windows, Version 24.0. Armonk, NY: IBM Corp.) was used for the statistical analyses. Results were considered statistically significant at p < 0.05. All parameters were tested for normality with a Shapiro – Wilk test.

As a preprocessing step, prior to further analyses, we tested all MRI variables in the control subject group for significant associations with age by using linear regression models, in order to detect dependency on physiological aging. Subsequently we applied the inverse transformation parameters for a mean age of 40 years to the MRI variable for both, control subject group and the patient group. This resulted in a complete reduction of age dependency in the control group, and a compensation of physiological aging in the patient group.

Group comparisons between CS and the patient subgroups regarding age and disease duration were assessed by univariate ANOVA with Games-Howell correction for multiple comparisons of post-hoc pairwise tests between the subgroups. For group differences of EDSS and global and ROI-derived quantitative MRI parameters (aMVF, R1, R2, PD) we applied Kruskal Wallis tests and corrected pairwise post- hoc tests for multiple comparisons with the Dunn–Bonferroni method correction. In order to provide an overview of the deviations of aMVF, R1, R2 or PD in the patient subgroups from CS we calculated standardized z-scores (z -scores relative to the control group) and summarized them in bar-plots. The individual z-scores were calculated as.

$$z=\frac{\mathrm{patient}\;\mathrm{result}-mean\;of\;CS\;group}{\mathrm{standard}\;\mathrm{deviation}\;of\;CS\;\mathrm{group}}$$

We evaluated associations between MUCCA and global as well as regional synthetic MR derived MVF and relaxometry results using Spearman rank correlation analyses. To account for the risk of false-positive findings due to multiple testing of correlations with MUCCA in 28 different atlas-based regions, we applied a Bonferroni – Holm correction for multiple comparisons.

In order to investigate whether iMVF, T2w lesion load or brain atrophy were most related to spinal cord atrophy, we used Least Absolute Shrinkage and Selection Operator (LASSO) regression with MUCCA as dependent variable and iMVF, brain parenchymal fraction and lesion load as independent variables. Lasso is a penalized regression technique that models the joint dependence of multiple variables on a quantitative parameter, and is suitable for variables tending to multicollinearity (Tibshirani, 1996). The obtained standardized coefficients were used to evaluate variable importance.

## 3 Results

### 3.1 Demographic, Clinical and global MRI data

Demographic and clinical data as well as global MRI parameters are depicted in Table 1. Of the 91 patients with MS, 49 (53.8 %) had RRMS, and 42 (46.2 %) had progressive MS, either SPMS (n = 27) or PPMS (n = 15). A total of 31 CS were included for group comparisons. Mean and median values of age, disease duration and EDSS were, as expected, higher in the PMS group in comparison with the RRMS group. MUCCA, total brain lesion load, as well as iMVF, brain GM and WM volumes as fractions of ICV (GMF, WMF) and brain parenchymal fraction (BPF) demonstrated statistic significant differences among CS, RRMS and PMS patients mostly at a p level of < 0.001. Intracranial MVF, BPF, GMF and WMF were significantly lower in the RRMS and moreover in the PMS patient group compared to CS, and significantly lower in PMS compared to RRMS. MUCCA was significantly lower in PMS compared to RRMS and to controls. Mean values of R1 and R2 relaxation rates averaged across the entire brain were smaller in RRMS and in PMS compared to the control group, and of R1 in PMS compared to RRMS. PD was merely increased in the PMS group compared to controls.

**Table 1:** Demographic and quantitative global MRI data.

	**Control subjects**	**RRMS**	**PMS**	**P-value**
**Number (% female)**	31 (63 %)	49 (55 %)	42 (57 %)	P = 0.070^$^
**EDSS (median [IQR])**	–	2.5 [1.5–2.5]	6.5 [6.0–7.0]	P < 0.001©
**Age (years)**	38.7 ± 11.8	43.5 ± 12	57.8 ± 6.4	P < 0.001^#,^©
**Disease duration (years)**	–	7.8 ± 7.6	17.9 ± 10.7	P < 0.001©
**Lesion load (ml)**	–	20.9 [11.2 – 20.7]	49.5 [36.8 – 86.8]	P < 0.001©
**MUCCA (mm^2^)**	82.6 [73.0 – 92.2]	75.0 [68.8 – 85.3]	58.0 [ 52.5 – 68.6]	P < 0.001^#,^©
**iMVF (%ICV)**	10.4 [9.6 – 10.7]	9.4 [8.9 – 10.2]	8.1 [7.5 – 8.7]	P < 0.001*^,#,^©
**White matter fraction (%ICV)**	35.3 [33.5 – 36.5]	33.0 [31.2 – 35.3]	30.2 [28.6 – 32.1]	P < 0.001*^,#,^©
**Gray matter fraction (%ICV)^a^**	50.3 [48.9 – 51.7]	49.0 [47.2 – 50.4	46.9 [44.3 – 46.1]	P < 0.001*^,#,^©
**Brain parenchymal fraction (BPV/ICV) ^a^**	87.2 [85.7 – 88.8]	83.3 [80.6 – 86.5]	78.4 [74.1 – 81.1]	P < 0.001*^,#,^©
**R1 in global brain (s^−1^)**	1.06 [1.04 – 1.08]	1.04 [1.02 – 1.06]	1.02 [0.99 – 1.03]	P < 0.001*^,#,^©
**R2 in global brain (s^−1^)**	11.8 [11.6 – 11.9]	11.6 [11.4 – 11.8]	11.4 [11.2 – 11.65]	P < 0.001*^,#^
**PD in global brain (%)**	78.7 [78.2 – 79.2]	78.8 [78.4 – 79.5]	79.3[78.5 – 79.9]	P < 0.001^#^

Age and disease duration presented as mean ± standard deviation, EDSS and quantitative MRI parameters reported as median [interquartile range].

Abbreviations: RRMS relapsing-remitting Multiple Sclerosis, PMS progressive Multiple Sclerosis, EDSS expanded disability status scale, IQR interquartile range, SD standard deviation, MUCCA normalized mean upper cervical cord area, iMVF intracranial myelin volume fraction, ICV intra-cranial volume, BPV brain parenchymal volume, R1 longitudinal relaxation rate, R2 transverse relaxation rate, PD proton density.

a corrected for physiological ageing.

$ Chi-square test.

Group differences for EDSS and quantitative MRI parameters assessed using Kruskal Wallis tests, for age and disease duration assessed using univariate ANOVA; if appropriate pairwise post-hoc tests corrected for multiple comparisons with Bonferroni – Holm and with Games-Howell correction respectively.

* P < 0.05 for pairwise comparison between CS and RRMS.

# P < 0.05 for pairwise comparison between CS and PMS.

© P < 0.05 for pairwise comparison between RRMS and PMS.

**Fig. 4:**
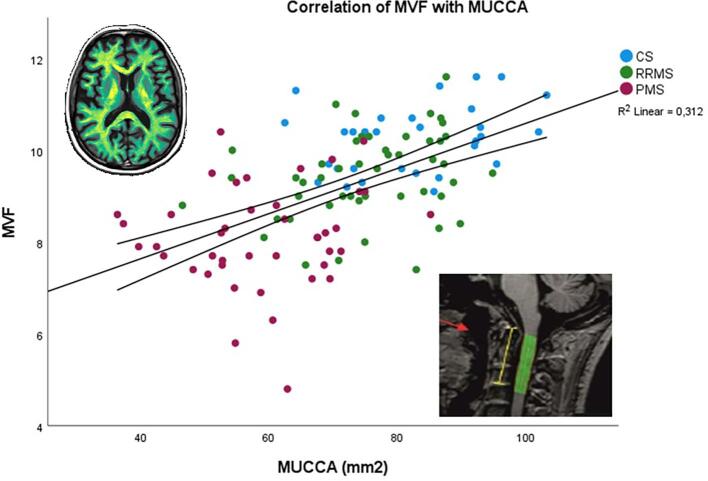
Scatter plot of the relationship between MUCCA and intracranial MVF in the whole group of study participants. Upper left: Myelin map derived from SyMRI Software. Down right: Estimation of MUCCA (NeuroQLab Software).

Among all MS patients, MUCCA was significantly associated with total brain lesion load, iMVF, GMF, WMF, and BPF by using Spearman rank correlation analyses (Table 2, Fig. 4.**)**. Therein the rho coefficients for correlation with MUCCA were greatest for iMVF, BPF and WMF. Separate Spearmann correlation analyses for RRMS and PMS patient groups yielded significant correlations of iMVF (ρ = 0.325), BPF (ρ = 0.293) and WMF (ρ = 0.349) with MUCCA only in the RRMS group (data not shown). The PMS group demonstrated no statistically significant results in this analysis.

**Table 2:** Spearmann correlations coefficients (ρ) between global MRI results and MUCCA for MS patients.

**global MRI results**	**MUCCA**
**Lesion load (ml)**	−0.489 (P < 0.001)
**iMVF (%ICV)**	0.591 (P < 0.001)
**White matter fraction (%ICV)**	0.573 (P < 0.001)
**Gray matter fraction (%ICV)^a^**	0.290 (P = 0.001)
**Brain parenchymal fraction (BPV/ICV)^a^**	0.552 (P < 0.001)

Abbreviations: MUCCA mean upper cervical cord area, iMVF intracranial myelin volume fraction, ICV intracranial volume, BPV brain parenchymal volume.

a: corrected for physiological ageing.

We analyzed which of the global MRI features had the highest impact on upper cervical cord atrophy in the entire patient group. By virtue of representing measures of global demyelination (including NAWM), focal lesion pathology, and brain atrophy, we chose iMVF, lesion load and BPF as variables. Potential outliers (especially 4 patients with lesion load > 140 ml) were excluded, also in order to succeed the normal distribution of the variable, resulting in 78 patients with all parameters available. A small degree of collinearity between these three parameters was obtained by variance inflation factors (VIF) of 1.8 for iMVF, 2.4 for lesion load and BPF as well as Pearson correlation coefficients of −0.63 for lesion load vs iMVF, −0.73 for lesion load vs BPF and 0.69 for iMVF vs BPF. Thus, we used LASSO regression, which is suitable for multicollinearity problems and we evaluated variable importance by use of standardized coefficients. The highest coefficient was obtained for iMVF (4.52). BPF and lesion load showed much lower coefficients of 0.37 and 0.003, respectively.

### 3.2 ROI analysis – Group comparisons

**Fig. 5:**
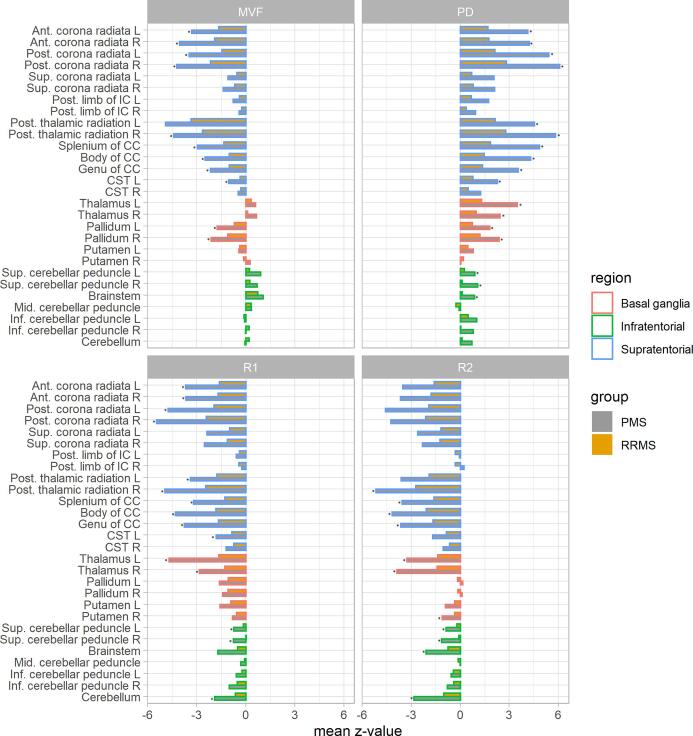
Normalized z-scores of aMVF, R1, R2 and PD within the selected brain ROIs (mean values) representing deviations of the patient subgroups from CS. Asterisks represent significant differences in quantitative MRI parameters between RRMS and PMS patients.

Median and interquartile ranges of the quantitative MRI parameters (aMVF, R1, R2 and PD) were assessed in all brain ROIs separately for CS and the two patient subgroups. The results are summarized as z-deviations compared to the control group in Fig. 5, and the detailed results of aMVF, R1, R2 and PD averaged in each ROI are shown in the supplement: Tables S1, S2 and S3.

In the control group the aMVF within the ROIs ranged between 22.4 % (body of corpus callosum) and 30.8 % (left anterior corona radiata) in the supratentorial regions, between 10.1 % (left putamen) and 20.2 % (thalamus) in the deep gray matter structures, and between 13.0 % (cerebellum) and 25.2 % (middle cerebellar peduncle) in the infratentorial regions. By comparison, lowest R1 and R2 and highest PD were determined in the cerebellum (R1 = 0.94 s^−1^, R2 = 11.0 s^−1^, PD = 82.6 %). Highest R1 and lowest PD were found in the anterior corona radiata (R1 = 1.60 s^−1^, PD = 65.4 %), while the pallidum was the structure with highest R2 in the control group (R2 = 15.6 s^−1^).

In the patient groups compared to CS we generally observed decreases of aMVF, R1 and R2 and elevations of PD in the ROIs.

Supratentorial white matter structures (Table S1 in supplementary material): in the anterior, posterior and superior corona radiata, posterior thalamic radiation and corpus callosum, we found significant group differences between the patient groups and CS regarding aMVF, relaxation rates and proton density. Differences between RRMS and PMS were significant for aMVF, R1 and PD in almost all of these ROIs, except for the superior corona radiata. R2 group differences between RRMS and PMS were significant for corpus callosum and right posterior thalamic radiation. In the left corticospinal tract, aMVF was reduced in PMS compared to CS and to RRMS. Furthermore in the CST, we detected R1 and R2 reductions and PD elevation predominantly in PMS in comparison to CS, while in the RRMS group only R1 reductions were observed in this structure. In the posterior limb of internal capsule, which partly belongs to the CST, lower aMVF, R1 and R2 in PMS compared to controls did not reach significance, while PD in the left hemisphere was significantly increased in PMS patients compared to controls.

Deep grey matter structures (DGM, Table S2 in supplementary material): among the investigated structures, the pallidum was the only region in which aMVF was significantly reduced in both patient groups compared to controls. In parallel, R1 and PD, but not R2, were significantly altered in both patient subgroups compared to CS. In the thalami, R1 and R2 were reduced and PD increased in RRMS and even more in PMS compared to CS, while aMVF was constant. In the putamen, alterations of R1, R2 and PD were mainly found in the PMS subgroup, at unchanged aMVF.

Infratentorial brain structures (Table S3 in supplementary material): within the brainstem and the superior cerebellar peduncles we observed increases of aMVF in PMS compared to controls, whereas aMVF was overall unchanged in the remaining infratentorial structures (cerebellum and middle and inferior cerebellar peduncles). By contrast, the corresponding relaxation rates R1, R2 showed lower values in the patient groups (PMS < RRMS < CS) and higher PD (PMS > RRMS > CS) in all of the infratentorial ROIs, similar to the findings in the supratentorial structures. Specifically, in the brainstem, cerebellum and in the superior cerebellar peduncles R1, R2 and PD were altered in PMS compared to controls and mostly in PMS compared with RRMS. In contrast, in the middle cerebellar peduncles we found no differences in R1, R2 or PD between the groups, while in the right inferior cerebellar peduncles these MRI parameters were altered in PMS compared to controls.

### 3.3 ROI analysis – Spearmann correlation analysis with MUCCA

Table 3 summarizes significant results (after correction for multiple comparisons) of the correlation analyses between MUCCA and ROI-based aMVF, R1, R2 and PD for the entire MS patient group. The corresponding correlation analysis for the CS group and separate analyses for the MS subgroups did not show any significant results.

**Table 3:** Spearmann correlations of MUCCA with SyMRI derived parameters (aMVF, R1, R2, PD) for MS patients.

**Correlation coefficient (P-value)**	**aMVF (%)**	**R1 (s^−1^)**	**R2(s^−1^)**	**PD (%)**
Anterior corona radiata R	0.352 (P = 0.027)	N.S.	N.S.	−0.375 (P = 0.006)
Posterior corona radiata R	0.335 (P = 0.027)	0.363 (P = 0.010)	N.S.	−0.349 (P = 0.021)
Posterior thalamic radiation (including optic radiation) R	0.358 (P = 0.027)	0.468 (P < 0.001)	0.326 (P = 0.048)	−0.493 (P < 0.001)
Body of corpus callosum	0.337 (P = 0.027)	0.465 (P < 0.001)	0.341 (P = 0.025)	−0.471 (P < 0.001)
Genu of Corpus Callosum	N.S.	0.385 (P = 0.004)	N.S.	−0.386 (P = 0.004)
Splenium of corpus callosum	*0.306 (P = 0.063)*	0.349 (P = 0.022)	N.S.	−0.410 (P < 0.001)
Brainstem	N.S.	0.353 (P = 0.022)	N.S.	N.S.
Cerebellum	N.S.	0.400 (P = 0.002)	0.438 (P < 0.001)	N.S.
Left Pallidum	0.327 (P = 0.044)	N.S.	N.S.	−0.342 (P = 0.021)
Right Thalamus	N.S	0.397 (P = 0.004)	0.417 (P = 0.0002)	−0.408 (P = 0.01)
Left Thalamus	N.S.	N.S.	0.343 (P = 0.015)	N.S.

Results are presented if P < 0.050 after Bonferroni – Holm correction for multiple comparisons. Abbreviations: MUCCA mean upper cervical cord area, aMVF average myelin volume fraction, R1 longitudinal relaxation rate, R2 transverse relaxation rate, PD proton density, N.S. not significant (P > 0.050).

In the MS patient group aMVF was significantly associated with MUCCA in supratentorial WM structures, like anterior and posterior corona radiata, posterior thalamic radiation, and body of corpus callosum as well as in the left pallidum. In accordance, R1 and PD (in the right anterior corona radiata and left pallidum only PD) demonstrated statistically significant associations with MUCCA in these structures. Brainstem, cerebellum, cerebellar peduncles, corticospinal tract, putamen, thalamus, posterior limb of internal capsule and superior corona radiata did not exhibit any significant associations between aMVF and MUCCA.

In contrast to aMVF, relaxation rates – especially R1 – presented significant associations with MUCCA not only in supratentorial but also in infratentorial structures, namely in the cerebellum and in the brainstem. Especially in the right thalamus relaxation rates and proton density correlated well with spinal cord atrophy measures. An additional subgroup analysis (data not shown) regarding RRMS and PMS separately showed no significant correlation between MUCCA and any of the four quantitative MRI parameters in both MS subgroups.

## 4 Discussion

Myelin damage occurs in the brain within inflammatory lesions or as diffuse demyelination of white and grey matter and is one of the major drivers of clinical disability in MS (Lassmann, 2018). In this study, we used the MRI sequence QRAPMASTER for fast quantification of whole brain and regional MVF, relaxation parameters R1, R2 and PD. Group comparisons were performed between large samples of relapsing and progressive MS patients as well as control subjects and demonstrated statistically significant alterations of intracranial and ROI-based MVF, relaxation rates and PD in patients with MS compared to controls. We addressed the question whether spinal cord atrophy is stronger related to global brain demyelination rather than to measures of focal lesion pathology or neurodegeneration. Global brain demyelination had a major impact on upper cervical cord atrophy in the MS group. On the regional scale, we demonstrated associations between cervical cord atrophy and infratentorial relaxation parameters, as well as between cord atrophy and aMVF, R1, R2 and PD in supratentorial ROIs.

### 4.1 Global quantitative MRI-Data and spinal cord atrophy

Intracranial MVF, WMF and GMF were reduced in relapsing as well as progressive MS compared to CS, with strongest effects in PMS (table 1). Our iMVF results matched well with a recent validation study using the same method that identified lower myelin fractions in the whole brain and NAWM of relapsing–remitting and progressive MS patients relative to healthy controls (Ouellette et al., 2020). That referenced study and other work demonstrated good correlations in the supratentorial brain between myelin quantification by QRAPMASTER and myelin related histological stains in ex-vivo examinations of brain specimens (Warntjes et al., 2017). Our BPF results were in close agreement with other studies using the same method to capture brain atrophy (Vågberg et al., 2013), or automatic or manual segmentation methods (Cappelle et al., 2020, Kassubek et al., 2003). Furthermore, brain grey and white matter fractions in our patient groups were consistent with previous studies in MS using fully-automated or semiautomatic methods for brain segmentation (Tedeschi et al., 2009, Jasek et al., 2020).

In accordance with the literature, we detected significant spinal cord atrophy in both patient groups compared to CS, which was most pronounced in PMS (table 1) (Casserly et al., 2018). We detected significant correlations between MUCCA and all assessed global brain MRI metrics, which were strongest for iMVF and BPF. Our LASSO regression model, relating MUCCA with measures of global brain demyelination, focal lesion pathology and neurodegeneration (iMVF, total lesion load and BPF) demonstrated the highest correlation coefficient for iMVF, indicating a relevant interaction of cerebral demyelination and atrophy in the upper spinal cord.

Aiming at the question whether the associations between MUCCA and iMVF were based on affection of structures that are functionally connected with the cervical cord, or whether they are caused by parallel but independent processes, we conducted a regional atlas-based quantification of aMVF, R1, R2 and PD and investigated their ROI-based associations with MUCCA.

### 4.2 Group differences of regional quantitative MR-Data between MS patient groups and control subjects.

We focused on brain ROIs which are functionally or structurally connected to the spinal cord, namely the corticospinal tracts and related structures (posterior part of the internal capsule, posterior corona radiata) the brainstem and cerebellum with the superior, inferior and middle cerebellar peduncles, and the corpus callosum as a highly myelinated reference structure.

Our aMVF results of the control group in the corpus callosum, internal capsule, corona radiata and posterior thalamic radiation (average MVF in ROIs between 22 % and 31 %) agreed with a recent ROI-based validation study on healthy controls that compared QRAPMASTER based myelin quantification with other MRI based methods (Hagiwara et al., 2018). Discrepancies of lower aMVF in some regions in our study might result from differences in the included CS groups, the applied atlases and details of the image postprocessing used to extract the quantitative parameters in the ROIs.

We demonstrated consistent decreases of aMVF, R1, and R2 as well as PD increase compared to CS in most of the selected supratentorial ROIs in RRMS and PMS patients, with the PMS group being more severely affected (Fig. 5 and supplementary table S1). The changes of aMVF in these regions corresponded well to the alterations of the relaxation rates and proton density, as could be expected based on the myelin model, by which aMVF is calculated on the basis of the R1, R2 and PD.

The corticospinal tracts showed most pronounced alterations of aMVF, R1, R2 and PD in the left CST in PMS compared to CS. The right CST was involved to a lesser extent and showed mainly reduced values of R1, R2 and higher PD in PMS compared to CS. Thus, the CST as a connecting structure between supratentorial and infratentorial regions was stronger affected in PMS than in RRMS, which supports the hypothesis that the tissue damage may spread downstream from supratentorial structures via the CST towards the spinal cord during the progression of the disease. Furthermore, a left–right hand side asymmetry of CST affection, especially in SPMS patients, is in line with a previous study that regarded multiparametric MRI indices (mean diffusivity, magnetization transfer ratio) (Reich et al., 2007). Similarly, in the posterior limb of the internal capsule, which is transected by the corticospinal tracts, alterations of aMVF, R1, R2 and PD between patients and controls were larger in the left hemisphere, though significant only for PD in the PPMS group. The relatively weak statistical effects in the posterior limb of the internal capsule compared to the entire CST, although a considerable part of its cross-sectional area belongs to the CST, could be due to the small size of this structure, which, together with the limited image resolution, contributes to an increased inter-individual variability of the MRI results.

In the DGM structures, basal ganglia and thalami (Fig. 5 and table S2 in supplementary material), we found significant differences between the controls and the patient groups mainly as R1 reduction and PD increase, and less as R2 decrease, but myelin reduction merely in the pallidum. Moreover, the thalamus, putamen and pallidum showed markedly different overall aMVF levels, with particularly low values of around 10 % aMVF in the putamen. These different patterns compared to the supratentorial ROIs may be interpreted by several factors which influence the myelin quantification in these DGM structures. GM generally contains only a small amount of myelin, which means that the aMVF results averaged in the ROI are overall lower than in the supratentorial WM. These structures each consist of a blend of white and grey matter, but may differ in their amount and structure of myelinated white and grey matter (Wolff and Vann, 2019, Kumar et al., 2021). Since the SyMRI myelin model (Warntjes et al., 2016) assumes a tissue structure with typical layered myelin sheaths, which is not or only partially valid in the DGM structures, the observable myelin in these structures may differ from the actual myelin content, and thus may also differ between the different, histologically complex DGM regions. Therefore we drew additional information about pathological changes in the DGM structures from the observed changes in R1, R2 and PD, because strong associations with myelin content have been shown for these parameters, especially for R1 and PD (Warntjes et al., 2016). We found significant alterations of R1 and PD in the MS groups in the thalami, putamen and pallidum, but unchanged and especially high R2 only in the latter structure. This finding was probably due to a high iron content in the pallidum (Hallgren and Sourander, 1958) in control subjects and moreover in patients with MS (Aquino et al., 2009, Khalil et al., 2011). It leads to shortening of the T2 relaxation times and consequently high R2, and may mask any R2 reduction due to demyelination or axonal degeneration. Overall we interpret the observed affection of the relaxation parameters in the DGM structures of our MS subgroups as indications of pathological processes, including myelin loss which were most pronounced in PMS.

When interpreting the aMVF in the infratentorial structures, similar issues as in the DGM emerged, related to MVF modelling in GM containing structures. The cerebellum and, to a lesser extent, the brainstem contain substantial amounts of GM, in the cerebellum in the form of the tightly folded cerebellar cortex and the dentate nucleus which make up more than half of the cerebellar volume, and in the brainstem by a number of GM nuclei intermingled with the WM fiber bundles. For the ROI containing the entire cerebellum, the aMVF therefore showed relatively low values (about 13 %), which, however, did not differ between the subgroups. By comparison, the aMVF in the middle cerebellar peduncles, which largely consist of the cerebellar WM fiber trunks, were significantly higher (approx. 25 %). In the brainstem we found an aMVF of about 19 %, which was even increased in the MS groups, significantly in PMS compared to the control group. The SyMRI myelin model may, as explained above, be only partly valid in the brainstem and the cerebellum because of their complex histopathologic structure of grey and white matter, and might not give a complete picture of myelin pathology. Therefore we referred to the relaxation parameters R1, R2 and PD for additional information on pathologic changes presumably including myelin loss in these structures. In the brainstem, the cerebellum and the superior cerebellar peduncles the observed R1 and R2 reductions and PD increases, especially in PMS compared to controls, corresponded to our hypothesis of a higher level of pathologic changes within these structures in the progressive MS forms and that in PMS there is a focus on downstream degradation of the infratentorial structures which include regions that belong to the descending corticospinal pathways. There are few previous studies that have examined relaxation parameters in infratentorial areas of the brain that could be used for direct comparison with our quantitative results. However, in our control group, we found good agreement between our R1 values in the brainstem and superior cerebellar peduncles and a recent study (Straub et al., 2019) that examined these structures based on MP2RAGE MRI sequences.

### 4.3 Associations between regional quantitative MR-data and upper cervical cord area

Associations between MUCCA and any ROI-derived aMVF, R1, R2 or PD were not found in the control group, showing that potential physiological associations between cervical cord volume and brain myelin or relaxation parameters were negligible in our study, and that the associations that we found in the patient groups were most probably related to the disease.

We detected correlations between aMVF and MUCCA in supratentorial WM structures (anterior and posterior corona radiata, posterior thalamic radiation, and body of corpus callosum) in MS patients. Herein, the anterior and posterior corona radiata contain motoric projection fibers that feed into the corticospinal tracts. The body of the corpus callosum is related with the motoric system by incorporating transversing interhemispheric fibers that originate in the bilateral motoric cortical areas. Demyelination in these structures can be a source of transsynaptic or anterograde Wallerian degeneration affecting the upper spinal cord. Recent studies which have shown that atrophy of the spinal cord in MS is more pronounced in the upper cervical cord than in the lower segments, and is mostly independent of local spinal cord lesions, have supported the hypothesis that cord atrophy might result from remote effects originating in the brain (Evangelou et al., 2005, Rocca et al., 2011). On the other hand, the association of MUCCA with regional supratentorial aMVF could also result from simultaneous, but independent diffuse demyelination in both regions as a manifestation of global disease activity.

In infratentorial structures and DGM, we observed correlations of MUCCA with relaxation rates and PD but hardly any association with aMVF (table 3). The identification of correlations between MUCCA and myelin in these structures may have been compromised by the effects explained in the previous section, which are related to the high proportion of GM in these ROIs limiting the validity of the SyMRI myelin model, and related to iron deposition the basal ganglia, which have an impact on the R2 relaxation rates.

Regarding the thalamus we observed correlations between MUCCA and R1, R2 and PD that were plausible when considering that the thalamus is directly connected to the spinal cord via the spinothalamic tracts, and thus, supports our hypothesis of associations between cord atrophy and pathological changes in functionally connected structures. Both, cervical cord and thalamus, have been shown to be highly sensitive to disease related volume loss and degradation of white matter fibers in MS (Hagström et al., 2017, Weeda et al., 2020, Zivadinov et al., 2013). Still, associations between alterations in these structures have rarely been investigated. One previous study using voxel-based morphometry has reported positive correlations between MUCCA and thalamic volume, as well as between MUCCA and brainstem and cerebellar volumes predominantly in RRMS (Bellenberg et al., 2015). We also identified positive correlations of MUCCA with R1 and R2 in the cerebellum and with R1 in the brainstem. Interpreting R1 and R2 decrease as unspecific indications of tissue degradation, these findings also agree with the cited voxel-based morphometric study (Bellenberg et al., 2015).

Furthermore, MUCCA was correlated with aMVF and proton density of the pallidum, but not with R1 or R2 in this structure. We assume that the large proportion of GM and predominant iron accumulation in the pallidum may mask associations of MUCCA with the relaxation rates.

Separate correlation analyses of relapsing and progressive MS did not detect significant associations of MUCCA with regional quantitative MR data in the brain, probably due to limited patient numbers in our subgroups. Still, we can assume that the associations between MUCCA and thalamic and infratentorial relaxation parameters in the entire MS group were probably driven by PMS, because alterations of R1, R2 and PD were strongest in PMS and previous studies have shown that microstructural involvement of the infratentorial brain is pronounced in progressive MS (Ceccarelli et al., 2009).

### 4.4 Limitations

We acknowledge the limitations of this study and the need to consider several technical and methodological issues. The QRAPMASTER sequence generates axial slices with 4 mm slice thickness and a voxel size of 4 mm^3^. Despite fair histological validations of supratentorial myelin estimation the SyMRI myelin model showed limitations in GM rich structures like the basal ganglia, brainstem and cerebellum, as described in the discussion. Furthermore, partial volume effects can have an impact on tissue classification in the SyMRI tissue compartment model at the boundaries between different tissue types or at interfaces between brain and CSF. Particularly in infratentorial structures, pulse and motion artifacts produced from vascular structures (transverse and sigmoid sinuses) or CSF can interfere with the relaxation process (McGowan and Patel, 2000, Chougar et al., 2020). Furthermore, susceptibility artifacts at air-tissue or bone-soft tissue interfaces (i.e. mastoid cells, sphenoid sinus) in *EPI*-based acquisitions, such as QRAPMASTER could also influence the estimation of myelin based on the SyMRI tissue model (Zhuo and Gullapalli, 2006) With regard to the ROI analysis we performed, imperfections in the ROIs-registration especially in small structures – in spite of carefully reviewing our MRI data – are able to influence the precision of our results. Moreover, we acknowledge that no lesion filling of the T1w images has been used before brain segmentation. This can affect the segmentation quality and consequently the estimated GM and WM volumes, because hypointense lesions could be misclassified as GM. Although we have not specifically addressed GM, for which lesion filling is particularly important, it is possible that the absence of lesion filling also affected our ROI-analysis to a certain degree. A general disease-related limitation of MRI-based myelin quantification methods is that they cannot separate immune-mediated demyelination from secondary loss of myelin as a direct consequence of axonal degeneration. Finally yet importantly, despite the fact that our study included a large number of patients with MS in the analysis using the QRAPMASTER technique compared to others, we acknowledge the fact that especially the number of progressive MS patients was limited with a small proportion of primary progressive MS.

## 5 Conclusions

In conclusion, global quantitative MR parameters including myelin volume fraction derived from QRAPMASTER sequence were suitable to detect differences between controls and MS patients as well as between relapsing and progressive MS. With regard to the upper spinal cord, the intracranial myelin volume fraction had the strongest impact on spinal cord volume, suggesting that cervical cord atrophy is influenced stronger by demyelination within the brain, than by neurodegeneration or inflammatory brain lesions. Regional investigation of brain quantitative MR parameters indicated more severe involvement of infratentorial structures in progressive MS disease than in RRMS. Associations between MUCCA and supratentorial myelin loss and thalamic and infratentorial R1, R2 and PD could be demonstrated in MS patients. Thus, quantification of myelin and relaxation parameters by QRAPMASTER sequence is suitable to detect disease specific differences in the brain in relapsing and progressive MS and can also contribute to the clarification of the origin of upper spinal cord atrophy in MS.

## CRediT authorship contribution statement

**Theodoros Ladopoulos:** Conceptualization, Methodology, Formal analysis, Investigation, Writing – original draft, Writing – review & editing, Visualization. **Britta Matusche:** Methodology, Formal analysis, Writing – original draft, Writing – review & editing, Visualization. **Barbara Bellenberg:** Conceptualization, Methodology, Formal analysis, Investigation, Writing – original draft, Writing – review & editing, Visualization. **Florian Heuser:** Formal analysis. **Ralf Gold:** Supervision, Validation, Funding acquisition. **Carsten Lukas:** Conceptualization, Methodology, Resources, Supervision, Validation. **Ruth Schneider:** Conceptualization, Methodology, Resources, Supervision, Validation, Funding acquisition.

## Declaration of Competing Interest

The authors declare that they have no known competing financial interests or personal relationships that could have appeared to influence the work reported in this paper.

## Data Availability

The data that has been used is confidential.
