# Supplementary material for: Relaxometry and brain myelin quantification with synthetic MRI in MS subtypes and their associations with spinal cord atrophy
Source: Neuroimage Clin. 2022 Aug 25;36:103166. doi: 10.1016/j.nicl.2022.103166 (PMC9463599; doi:10.1016/j.nicl.2022.103166)
Supplement: Supplementary data 1 [file mmc1.docx]

**Supplementary Material: „Relaxometry and brain myelin quantification with synthetic MRI in MS subtypes and their associations with spinal cord atrophy“**

| **Supratentorial white matter structures** | median [IQR] | Patient group | MVF (%) | R1 (s^-1^) | R2 (s^-1^) | PD (%) |
| --- | --- | --- | --- | --- | --- | --- |
|  | **Anterior corona**  **radiata L ^a^** | CS | 30.5 [29.5 - 31.5] ^*, #^ | 1.59 [1.57 - 1.64]^*, #^ | 13.7 [13.5 - 13.9]^**, #^ | 65.7 [65.0 - 66.6]^*,#^ |
|  |  | RRMS | 28.8 [27.4 - 29.8]^©^ | 1.56 [1.49 - 1.58]^©^ | 13.2 [12.9 - 13.8] | 66.8 [66.1 - 67.9]^©^ |
|  |  | PMS | 26.8 [23.9 - 29.3] | 1.48 [1.33 - 1.54] | 12.7 [11.7 - 13.5] | 68.4 [66.9 - 70.9] |
|  | **Anterior corona**  **radiata R ^a^** | CS | 30.8 [29.8 - 31.8]^*, #^ | 1.60 [1.56 - 1.64]^*, #^ | 13.7 [13.4 - 13.9]^*, #^ | 65.4 [64.6 - 66.3]^*, #^ |
|  |  | RRMS | 28.6 [26.7 - 30.0]^©^ | 1.54 [1.47 - 1.57]^©^ | 13.1 [12.7 - 13.6] | 66.8 [66.0 - 68.2]^©^ |
|  |  | PMS | 25.9 [22.8 - 28.4] | 1.46 [1.30 - 1.53] | 12.7 [11.4 - 13.4] | 69.1 [67.4 - 72.2] |
|  | **Posterior corona**  **radiata L ^a^** | CS | 27.5 [26.4 - 28.6]^*, #^ | 1.49 [1.46 - 1.52]^*, #^ | 12.4 [12.1 - 12.5]^*, #^ | 67.7 [67.0 - 68.4]^*, #^ |
|  |  | RRMS | 26.0 [24.7 - 27.3]^©^ | 1.44 [1.36 - 1.47]^©^ | 11.9 [11.4 - 12.2] | 68.9 [67.8 - 70.1]^©^ |
|  |  | PMS | 23.6 [20.0 - 25.7] | 1.35 [1.13 - 1.40] | 11.3 [10.1 - 11.9] | 71.1 [69.1 - 74.6] |
|  | **Posterior corona**  **radiata R ^a^** | CS | 27.8 [26.7 - 28.6]^*, #^ | 1.49 [1.46 - 1.53]^*, #^ | 12.4 [12.2 - 12.6]^*, #^ | 67.6 [67.1 - 68.3]^*, #^ |
|  |  | RRMS | 25.2 [23.6 - 27.3]^©^ | 1.41 [1.35 - 1.48]^©^ | 11.9 [11.3 - 12.4] | 69.4 [67.8 - 70.9]^©^ |
|  |  | PMS | 23.2 [20.7 - 25.1] | 1.32 [1.21 - 1.40] | 11.4 [10.4 - 12.0] | 71.5 [69.2 - 75.1] |
|  | **Superior corona**  **radiata L ^a^** | CS | 26.6 [25.4 - 27.9]^#^ | 1.45 [1.41 - 1.49]^#^ | 12.3 [12.2 - 12.7]^*,#^ | 68.3 [67.4 - 69.0]^#^ |
|  |  | RRMS | 26.4 [24.3 - 27.4] | 1.42 [1.36 - 1.46] | 12.2 [11.7 - 12.4] | 68.4 [67.8 - 70.0] |
|  |  | PMS | 25.1 [23.0 - 27.4] | 1.36 [1.20 - 1.44] | 11.9 [10.5 - 12.4] | 69.3 [68.0 - 73.6] |
|  | **Superior corona**  **radiata R ^a^** | CS | 26.7 [25.4 - 27.9]^*, #^ | 1.45 [1.41 - 1.48]^*, #^ | 12.3 [12.2 - 12.7]^*, #^ | 68.2 [67.4 - 68.9]^*, #^ |
|  |  | RRMS | 25.8 [24.8 - 26.5] | 1.40 [1.35 - 1.43] | 12.1 [11.8 - 12.3] | 68.8 [68.3 - 69.7] |
|  |  | PMS | 24.7 [23.1 - 26.0] | 1.33 [1.24 - 1.42] | 11.8 [11.3 - 12.3] | 69.7 [68.5 - 72.1] |
|  | **Posterior limb of IC L ^a^** | CS | 28.9 [27.4 - 30.2] | 1.42 [1.36 - 1.44] | 12.9 [12.7 - 13.3] | 65.5 [64.9 - 66.4]^#^ |
|  |  | RRMS | 28.3 [27.2 - 29.4] | 1.40 [1.36 - 1.43] | 13.0 [12.7 - 13.1] | 66.1 [65.6 - 67.2] |
|  |  | PMS | 27.3 [25.6 - 29.3] | 1.37 [1.33 - 1.43] | 12.9 [12.5 - 13.4] | 67.0 [65.9 - 68.2] |
|  | **Posterior limb of IC R ^a^** | CS | 28.6 [26.7 - 30.0] | 1.41 [1.36 - 1.43] | 13.0 [12.7 - 13.2] | 66.1 [65.2 - 66.8] |
|  |  | RRMS | 28.1 [27.2 - 28.9] | 1.39 [1.35 - 1.42] | 13.0 [12.5 - 13.1] | 66.4 [65.5 - 67.1] |
|  |  | PMS | 27.8 [26.3 - 29.4] | 1.37 [1.35 - 1.43] | 13.0 [12.6 - 13.3] | 66.7 [66.1 - 67.6] |
|  | **Posterior thalamic radiation (including optic radiation) L ^a^** | CS | 30.6 [29.8 - 31.3]^*,#^ | 1.57 [1.51 - 1.61]^*,#^ | 12.9 [12.4 - 13.2]^*,#^ | 66.4 [65.9 - 67.1]^*,#^ |
|  |  | RRMS | 26.8 [24.0 - 29.6] | 1.44 [1.37 - 1.55]^©^ | 12.0 [11.4 - 12.5] | 69.0 [66.9 - 71.1]^©^ |
|  |  | PMS | 25.7 [21.8 - 29.1] | 1.38 [1.18 - 1.49] | 11.4 [10.1 - 12.4] | 70.9 [68.5 - 75.6] |
|  | **Posterior thalamic radiation (including optic radiation) R ^a^** | CS | 30.7 [29.7 - 31.6]^*,#^ | 1.60 [1.54 - 1.63]^*,#^ | 13.2 [12.8 - 13.4]^*,#^ | 65.8 [65.0 - 66.2]^*,#^ |
|  |  | RRMS | 27.5 [23.6 - 29.8]^©^ | 1.46 [1.37 - 1.53]^©^ | 12.2 [11.6 - 12.9]^©^ | 68.4 [67.1 - 71.0]^©^ |
|  |  | PMS | 23.9 [21.4 - 27.4] | 1.30 [1.16 - 1.39] | 11.6 [10.3 - 12.1] | 73.1 [69.5 - 75.4] |
|  | **Splenium of corpus callosum ^a^** | CS | 29.8 [28.7 - 30.5]^*,#^ | 1.51 [1.46 - 1.56]^*,#^ | 13.0 [12.8 - 13.2]^*,#^ | 66.6 [65.9 - 67.3]^*,#^ |
|  |  | RRMS | 28.2 [26.8 - 29.8]^©^ | 1.44 [1.39 - 1.48]^©^ | 12.6 [12.2 - 12.8]^©^ | 68.1 [66.8 - 69.2]^©^ |
|  |  | PMS | 26.3 [23.9 - 28.4] | 1.34 [1.20 - 1.44] | 11.9 [11.1 - 12.6] | 70.1 [68.4 - 74.3] |
|  | **Body of corpus**  **callosum ^a^** | CS | 22.4 [21.1 - 22.9]^*,#^ | 1.31 [1.26 - 1.33]^*,#^ | 12.3 [12.0 - 12.4]^*,#^ | 72.2 [71.9 - 73.4]^*,#^ |
|  |  | RRMS | 21.2 [19.8 - 22.3]^©^ | 1.23 [1.19 - 1.27]^©^ | 11.7 [11.4 - 11.9]^©^ | 73.5 [72.7 - 74.8]^©^ |
|  |  | PMS | 19.2 [18.0 - 20.8] | 1.12 [1.05 - 1.17] | 11.1 [10.6 - 11.5] | 76.2 [74.4 - 78.1] |
|  | **Genu of Corpus Callosum ^a^** | CS | 28.7 [27.9 - 30.0]^*,#^ | 1.46 [1.42 - 1.50]^*,#^ | 13.0 [12.8 - 13.4]^*,#^ | 68.9 [68.2 - 70.2]^*,#^ |
|  |  | RRMS | 27.5 [26.2 - 28.8]^©^ | 1.38 [1.31 - 1.44]^©^ | 12.4 [12.0 - 12.9]^©^ | 70.7 [69.6 - 72.0]^©^ |
|  |  | PMS | 26.2 [24.5 - 27.6] | 1.26 [1.16 - 1.34] | 11.5 [10.5 - 12.2] | 73.6 [71.6 - 75.8] |
|  | **Left CST** | CS | 24.1 [23.4 - 25.3]^#^ | 1.32 [1.29 - 1.36]^*,#^ | 12.2 [12.0 - 12.5]^#^ | 69.3 [68.8 - 69.8]^#^ |
|  |  | RRMS | 24.1 [23.2 - 24.9]^©^ | 1.29 [1.25 - 1.33]^©^ | 12.1 [11.9 - 12.3] | 69.7 [69.2 - 70.5]^©^ |
|  |  | PMS | 22.9 [21.0 - 24.4] | 1.24 [1.20 - 1.30] | 11.8 [11.4 - 12.2] | 71.1 [69.7 - 72.5] |
|  | **Right CST** | CS | 24.4 [23.3 - 25.2] | 1.32 [1.30 - 1.35]^*,#^ | 12.2 [12.0 - 12.5]^#^ | 69.4 [68.8 - 69.8]^#^ |
|  |  | RRMS | 23.7 [22.8 - 24.7] | 1.29 [1.26 - 1.33] | 12.1 [11.9 - 12.4] | 69.6 [69.1 - 70.2] |
|  |  | PMS | 23.5 [22.3 - 24.6] | 1.26 [1.21 - 1.32] | 11.9 [11.6 - 12.3] | 70.3 [69.6 - 71.1] |

**Table S1.** Group comparisons for MVF, R1 and R2 relaxation rates and PD (presented as median [IQR]): supratentorial white matter structures. Abbreviations: IQR interquartile range, MVF Myelin volume fraction, R1 longitudinal relaxation rate, R2 transverse relaxation rate, PD proton density, CSCS control subjects, RRMS relapsing remitting multiple sclerosis, PMS progressive multiple sclerosis; a: the quantitative MRI parameters were corrected for physiological ageing. Group Comparisons (CS, RRMS, PMS) using Kruskal-Wallis tests with post-hoc pairwise comparisons of subgroups (Dunn-Bonferoni tests), * P<0.05 for pairwise comparison between CS and RRMS, # P<0.05 for pairwise comparison between CS and PMS, © P<0.05 for pairwise comparison between RRMS and PMS.

| **Deep gray matter structures** | **median [IQR]** | **Patient group** | **MVF (%)** | **R1 (s^-1^)** | **R2 (s^-1^)** | **PD (%)** |
| --- | --- | --- | --- | --- | --- | --- |
|  | **Left Thalamus^a^** | CS | 15.3 [14.4 – 15.6] | 1.14 [1.10 - 1.17]^*,#^ | 12.4 [12.2 – 12.5]^*,#^ | 77.0 [76.6 – 77.5]^*,#^ |
|  |  | RRMS | 14.9 [14.2 – 16,5] | 1.08 [1.03 - 1.13]^©^ | 11.9 [11.5 - 12.6]^©^ | 78.1 [77.0 – 79.2]^©^ |
|  |  | PMS | 15.6 [14.2 – 17.2] | 1.01 [0.94 – 1.06] | 10.9 [10.1 – 11.8] | 79.3 [77.9 – 80.9] |
|  | **Right Thalamus^a^** | CS | 14.1 [13.4 – 15.3] | 1.13 [1.08 - 1.15]^*,#^ | 12.4 [12.2 – 12.7]^*,#^ | 77.7 [76.9 – 78.4]^*,#^ |
|  |  | RRMS | 14.0 [13.2 – 15.4] | 1.06 [1.01 - 1.10]^©^ | 12.0 [11.3 – 12.4]^©^ | 78.8 [77.7 – 79.7]^©^ |
|  |  | PMS | 14.9 [13.3 – 17.0] | 0.99 [0.92 – 1.04] | 10.8 [9.8 – 11.6] | 80.2 [78.9 – 81.6] |
|  | **Left Pallidum** | CS | 19.2 [17.3 - 20.6]^*,#^ | 1.37 [1.34 - 1.42]^*,#^ | 15.6 [15.1 - 15.8] | 72.9 [71.9 - 74.1]^*,#^ |
|  |  | RRMS | 17.7 [15.7 - 19.0]^©^ | 1.35 [1.28 - 1.38] | 15.5 [15.0 - 15.8] | 73.9 [73.0 - 75.3]^©^ |
|  |  | PMS | 15.3 [13.6 - 16.2] | 1.30 [1.26 - 1.35] | 15.6 [15.1 - 16.1] | 75.7 [75.0 - 76.7] |
|  | **Right Pallidum** | CS | 21.0 [20.0 - 22.9]^*,#^ | 1.40 [1.35 - 1.44]^*,#^ | 15.3 [14.7 - 15.5] | 71.7 [70.6 - 72.3]^*,#^ |
|  |  | RRMS | 19.2 [17.9 - 20.8]^©^ | 1.34 [1.29 - 1.38] | 15.1 [14.9 - 15.4] | 73.0 [71.9 - 73.8]^©^ |
|  |  | PMS | 18.0 [16.6 - 19.7] | 1.31 [1.27 - 1.37] | 15.3 [14.8 - 15.7] | 73.9 [72.7 - 74.9] |
|  | **Left Putamen ^a^** | CS | 10.1 [8.8 - 11.5] | 1.16 [1.14 - 1.19]^*,#^ | 13.7 [13.5 - 14.2]^#^ | 79.1 [78.3 - 80.2]^#^ |
|  |  | RRMS | 10.0 [8.9 - 10.5] | 1.13 [1.11 - 1.15] | 13.7 [13.5 - 13.9] | 79.7 [78.8 - 80.4] |
|  |  | PMS | 9.6 [8.6 - 10.8] | 1.12 [1.07 - 1.13] | 13.4 [13.0 - 13.9] | 79.9 [79.0 - 81.1] |
|  | **Right Putamen ^a^** | CS | 10.6 [9.4 - 12.0] | 1.16 [1.13 - 1.19]^#^ | 13.9 [13.6 - 14.1]^#^ | 78.9 [78.2 - 79.6] |
|  |  | RRMS | 10.3 [9.2 - 11.3] | 1.14 [1.11 - 1.17] | 13.7 [13.4 - 13.9]^©^ | 79.1 [78.3 - 79.9] |
|  |  | PMS | 11.0 [10.0 - 11.6] | 1.12 [1.08 - 1.17] | 13.4 [12.9 - 13.6] | 79.1 [78.1 - 80.0] |

**Table S2**. Group comparisons for MVF, R1 and R2 relaxation rates and PD (presented as median [IQR]): deep grey matter structures. Abbreviations: IQR interquartile range, MVF Myelin volume fraction, R1 longitudinal relaxation rate, R2 transverse relaxation rate, PD proton density, CSCS control subjects, RRMS relapsing remitting multiple sclerosis, PMS progressive multiple sclerosis; a: the quantitative MRI parameters were corrected for physiological ageing. Group Comparisons (CS, RRMS, PMS) using Kruskal-Wallis tests with post-hoc pairwise comparisons of subgroups (Dunn-Bonferoni tests). * P<0.05 for pairwise comparison between CS and RRMS, # P<0.05 for pairwise comparison between CS and PMS, © P<0.05 for pairwise comparison between RRMS and PMS.

| **Infraterntorial structures** | median [IQR] | Patient group | MVF (%) | R1 (s^-1^) | R2 (s^-1^) | PD (%) |
| --- | --- | --- | --- | --- | --- | --- |
|  | **Brainstem** | CS | 19.0 [18.2 - 19.7]^#^ | 1.13 [1.12 - 1.17]^*,#^ | 11.5 [11.3 - 11.7]^#^ | 74.9 [74.1 - 75.7]^#^ |
|  |  | RRMS | 19.4 [18.8 - 20.8] | 1.11 [1.09 - 1.15] | 11.4 [11.1 - 11.5]^©^ | 75.1 [74.5 - 75.8]^©^ |
|  |  | PMS | 19.9 [18.8 - 20.5] | 1.08 [1.04 - 1.11] | 10.9 [10.6 - 11.4] | 75.8 [74.8 - 76.6] |
|  | **Superior cerebellar peduncle L ^a^** | CS | 17.3 [16.2 - 19.1]^#^ | 0.99 [0.93 - 1.01]^#^ | 10.2 [9.7 - 10.6]^#^ | 74.6 [72.7 - 76.0]^#^ |
|  |  | RRMS | 17.9 [16.7 - 19.1] | 0.95 [0.91 - 0.99]^©^ | 9.9 [9.5 - 10.4]^©^ | 75.2 [73.6 - 76.8]^©^ |
|  |  | PMS | 18.9 [17.5 - 21.1] | 0.92 [0.86 - 0.96] | 9.5 [8.9 - 10.1] | 76.6 [74.8 - 78.3] |
|  | **Superior cerebellar peduncle R ^a^** | CS | 17.1 [15.4 - 18.9]^#^ | 0.98 [0.93 - 1.02]^#^ | 10.4 [10.0 - 10.7]^#^ | 74.4 [73.3 - 76.0]^#^ |
|  |  | RRMS | 17.7 [16.4 - 19.0] | 0.96 [0.92 - 1.01]^©^ | 10.4 [9.9 - 10.7]^©^ | 74.9 [74.0 - 76.0]^©^ |
|  |  | PMS | 18.9 [16.7 - 20.1] | 0.91 [0.86 - 0.96] | 9.6 [9.2 - 10.1] | 76.6 [74.6 - 78.9] |
|  | **Middle cerebellar peduncle** | CS | 25.2 [24.1 - 26.0] | 1.38 [1.34 - 1.40] | 12.5 [12.4 - 12.7] | 69.5 [68.9 - 69.9] |
|  |  | RRMS | 25.4 [24.5 - 26.5] | 1.37 [1.34 - 1.41] | 12.5 [12.3 - 12.7] | 69.2 [68.5 - 70.0] |
|  |  | PMS | 25.5 [24.7 - 26.8] | 1.37 [1.33 - 1.40] | 12.6 [12.3 - 12.8] | 69.2 [68.5 - 70.1] |
|  | **Left inferior cerebellar peduncle** | CS | 20.6 [18.9 - 22.0] | 1.19 [1.16 - 1.22] | 11.7 [11.5 - 12.1] | 71.7 [70.3 - 72.7]^#^ |
|  |  | RRMS | 20.1 [18.8 - 21.8] | 1.19 [1.15 - 1.22] | 11.8 [11.5 - 12.0] | 72.8 [71.2 - 73.9] |
|  |  | PMS | 20.3 [18.6 - 21.4] | 1.17 [1.12 - 1.20] | 11.7 [11.1 - 12.0] | 73.6 [72.2 - 75.0] |
|  | **Right inferior cerebellar peduncle** | CS | 19.6 [18.4 - 21.0] | 1.16 [1.14 - 1.20]^#^ | 11.8 [11.5 - 12.1]^#^ | 72.5 [71.4 - 73.7]^#^ |
|  |  | RRMS | 20.1 [18.5 - 21.3] | 1.14 [1.10 - 1.17] | 11.7 [11.3 - 11.9] | 73.0 [71.3 - 73.9] |
|  |  | PMS | 19.7 [17.8 - 21.5] | 1.10 [1.06 - 1.15] | 11.4 [11.0 - 11.8] | 73.8 [72.3 - 75.7] |
|  | **Cerebellum** | CS | 13.0 [12.5 - 13.4] | 0.94 [0.91 - 0.96]^#^ | 11.0 [10.9 - 11.3]^*,#^ | 82.6 [82.2 - 83.3] ^#^ |
|  |  | RRMS | 13.2 [12.5 - 13.8] | 0.91 [0.89 - 0.94]^©^ | 10.8 [10.5 - 11.0]^©^ | 82.7 [82.2 - 83.4] |
|  |  | PMS | 13.0 [12.4 - 13.7] | 0.88 [0.84 - 0.91] | 10.3 [9.9 - 10.6] | 83.1 [82.8 - 84.4] |

**Table S3**. Group comparisons for MVF, R1 and R2 relaxation rates and PD (presented as median [IQR]): infratentorial structures. Abbreviations: IQR interquartile range, MVF Myelin volume fraction, R1 longitudinal relaxation rate, R2 transverse relaxation rate, PD proton density, CSCS control subjects, RRMS relapsing remitting multiple sclerosis, PMS progressive multiple sclerosis; a: the quantitative MRI parameters were corrected for physiological ageing. Group Comparisons (CS, RRMS, PMS) using Kruskal-Wallis tests with post-hoc pairwise comparisons of subgroups (Dunn-Bonferoni tests). * P<0.05 for pairwise comparison between CS and RRMS, # P<0.05 for pairwise comparison between CS and PMS, © P<0.05 for pairwise comparison between RRMS and PMS


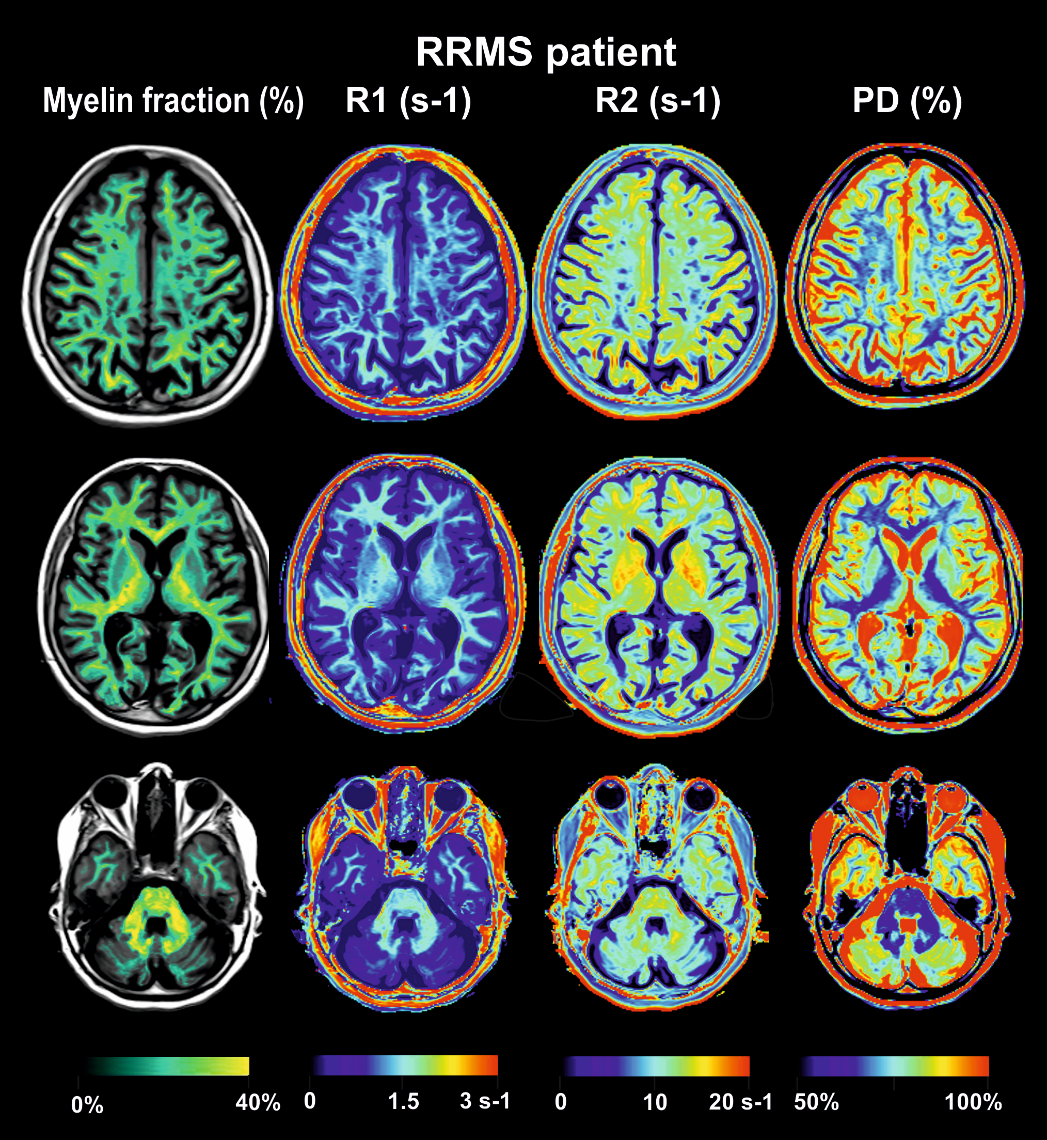


**Figure S1.** SyMRI-derived maps of relaxations rates R1 and R2 (A,B), proton density(C) and myelin (D) of a female RRMS patient.
